# Supplementary figures and images for: Mechanical stretching of pulmonary vein stimulates matrix metalloproteinase-9 and transforming growth factor-β1 through stretch-activated channel/MAPK pathways in pulmonary hypertension due to left heart disease model rats
Source: PLoS One. 2020 Sep 3;15(9):e0235824. doi: 10.1371/journal.pone.0235824 (PMC7470280; doi:10.1371/journal.pone.0235824)

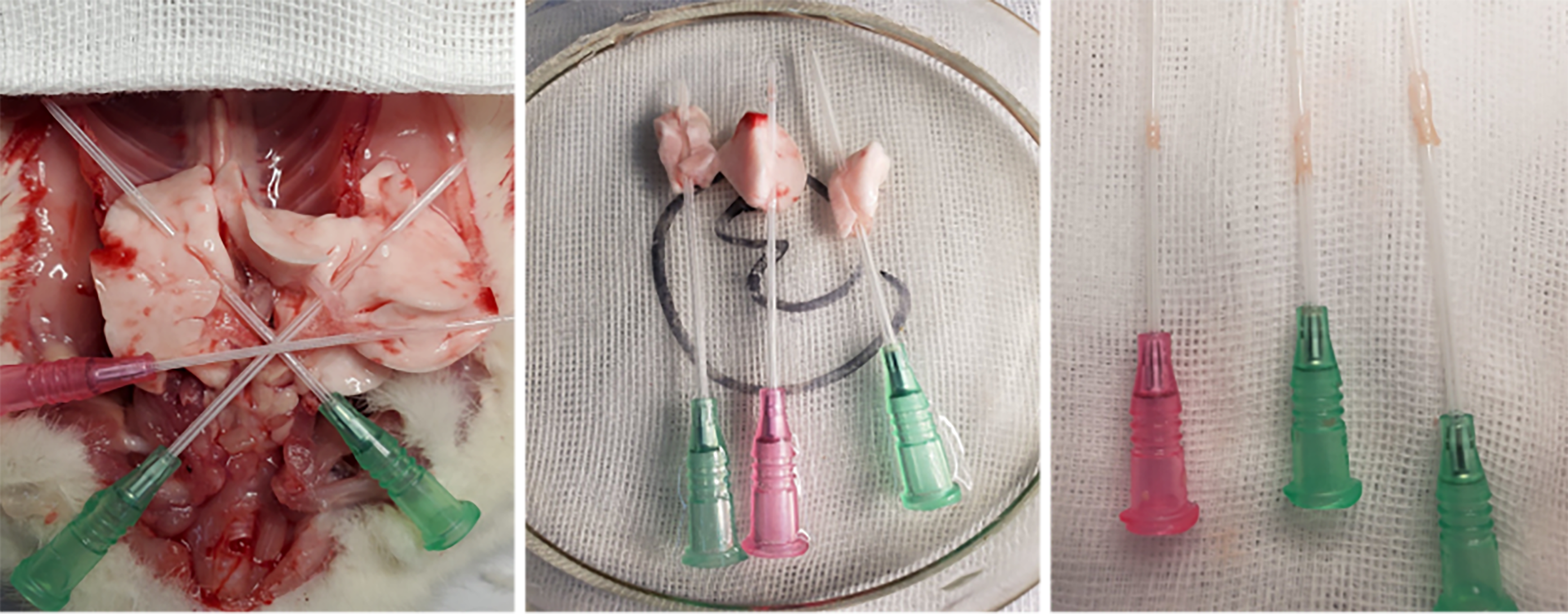

Supplement: S1 Fig — (TIF) [file pone.0235824.s001.tif]

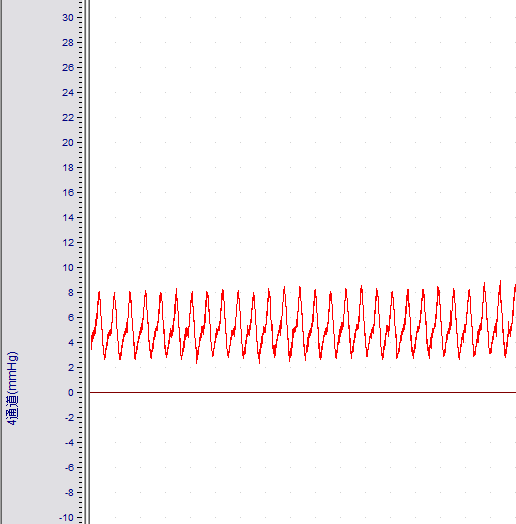

Supplement: S1 Raw data — (ZIP) [file pone.0235824.s002.zip › Raw data/Figure3 B1.PNG]

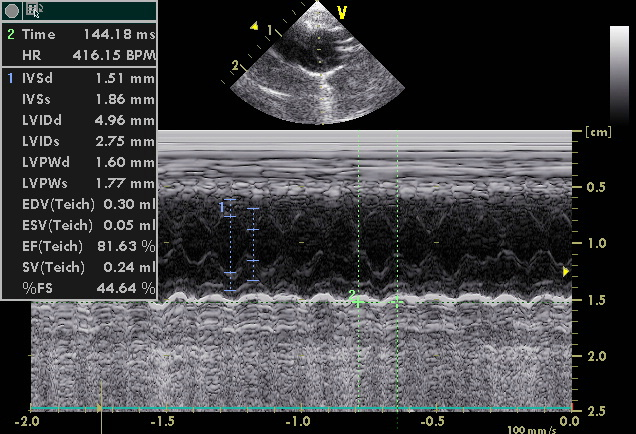

Supplement: S1 Raw data — (ZIP) [file pone.0235824.s002.zip › Raw data/Figure2 A1.tif]

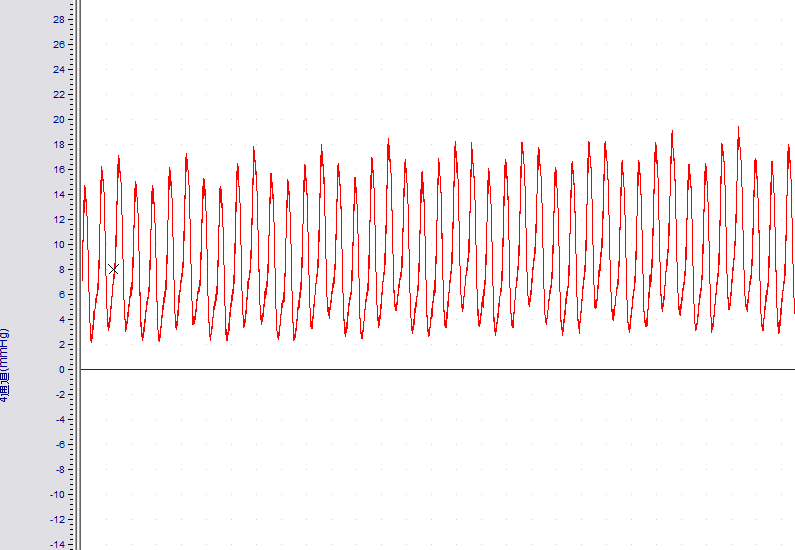

Supplement: S1 Raw data — (ZIP) [file pone.0235824.s002.zip › Raw data/Figure3 B2.PNG]

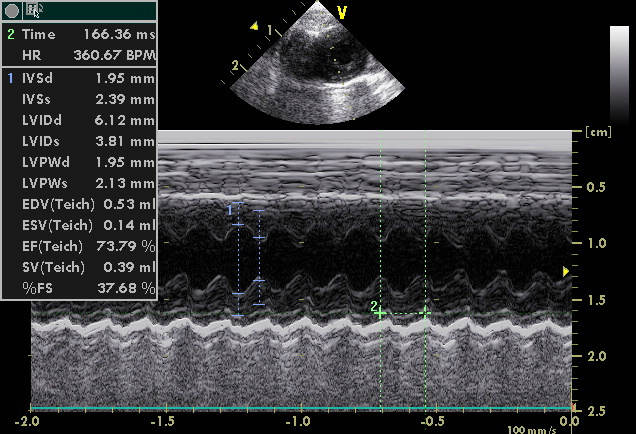

Supplement: S1 Raw data — (ZIP) [file pone.0235824.s002.zip › Raw data/Figure2 A2.tif]

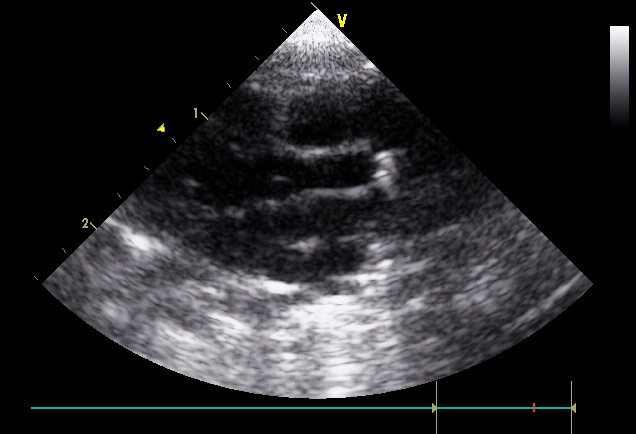

Supplement: S1 Raw data — (ZIP) [file pone.0235824.s002.zip › Raw data/Figure1 B2.tif]

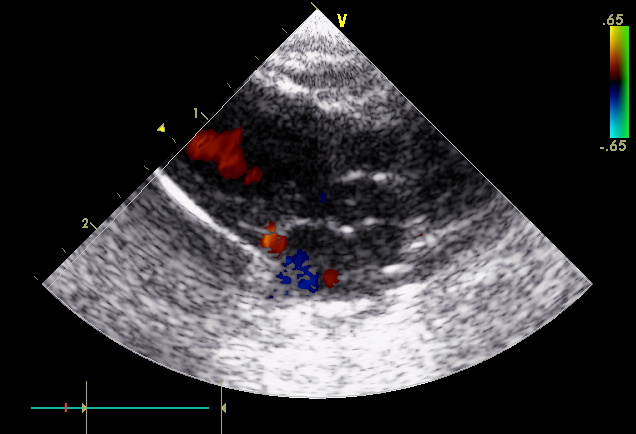

Supplement: S1 Raw data — (ZIP) [file pone.0235824.s002.zip › Raw data/Figure1 B1.tif]

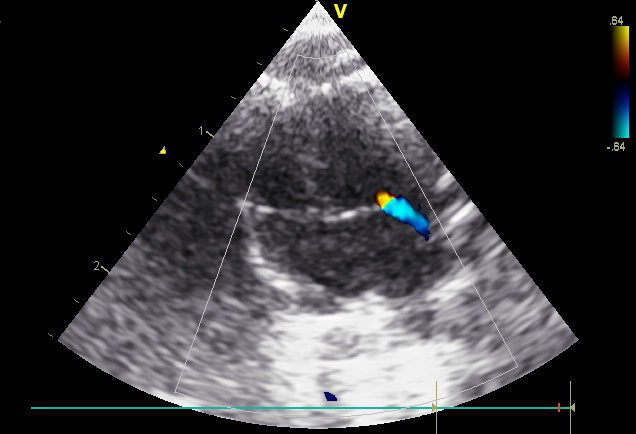

Supplement: S1 Raw data — (ZIP) [file pone.0235824.s002.zip › Raw data/Figure2 B1.png]

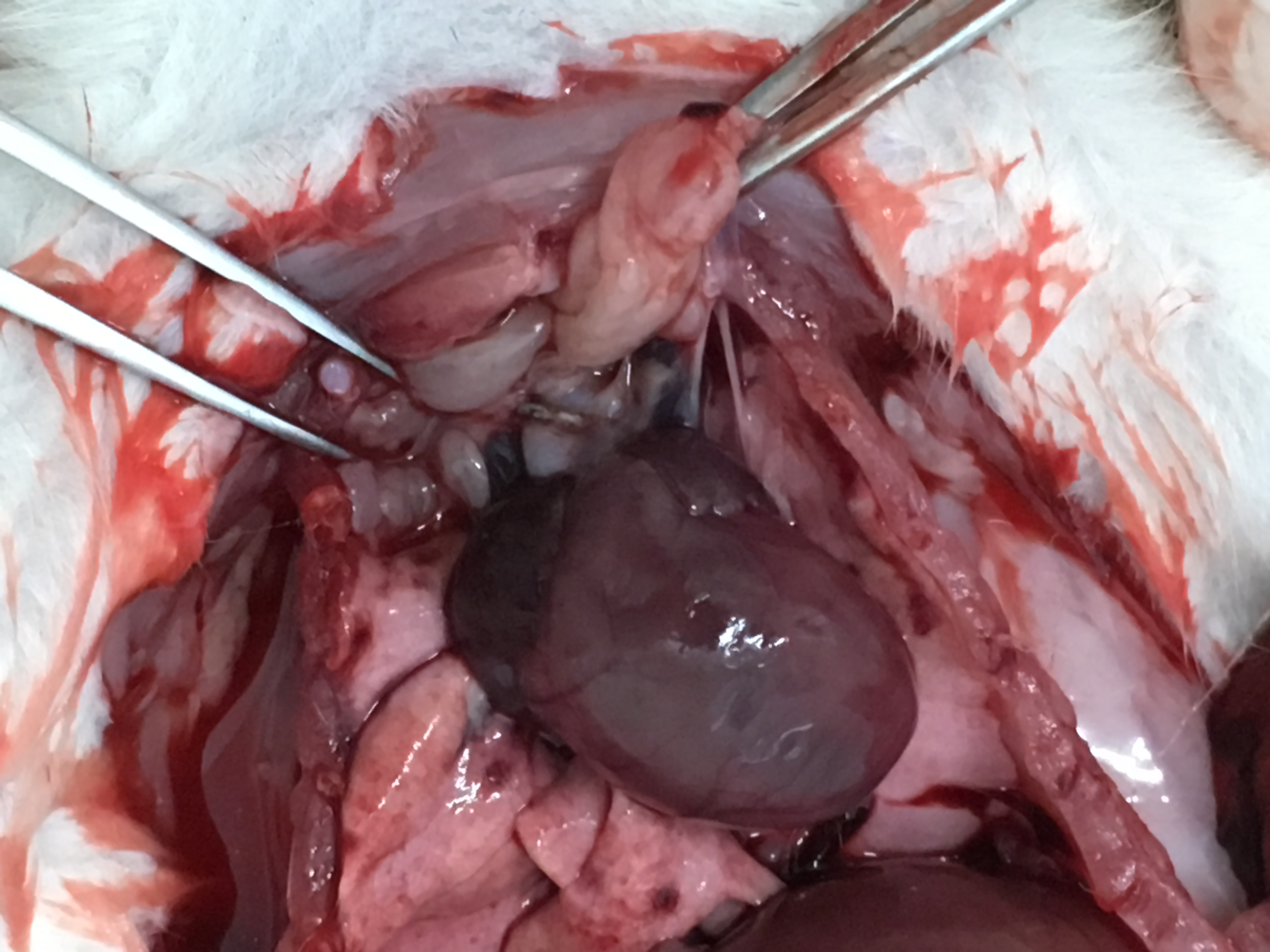

Supplement: S1 Raw data — (ZIP) [file pone.0235824.s002.zip › Raw data/Figure1 A.jpg]

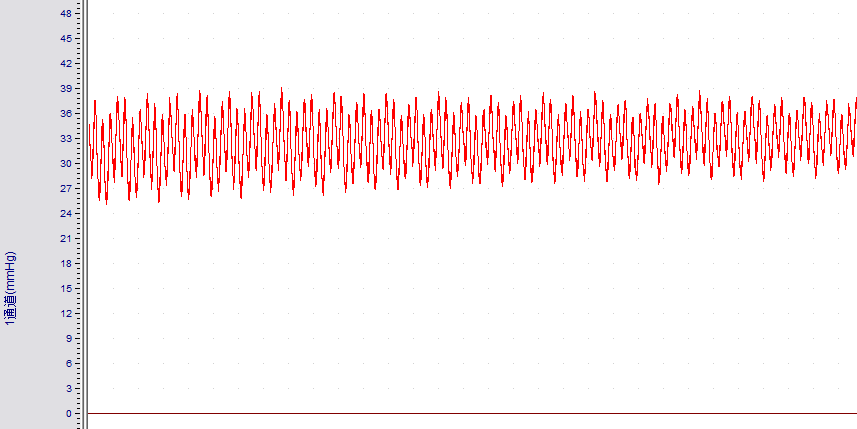

Supplement: S1 Raw data — (ZIP) [file pone.0235824.s002.zip › Raw data/Figure3 A2.png]

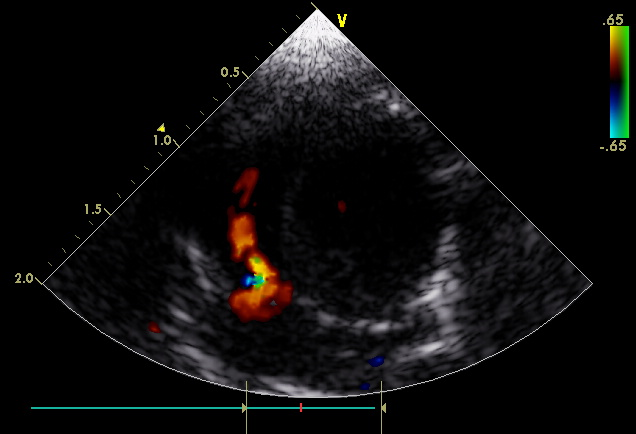

Supplement: S1 Raw data — (ZIP) [file pone.0235824.s002.zip › Raw data/Figure2 B2.tif]

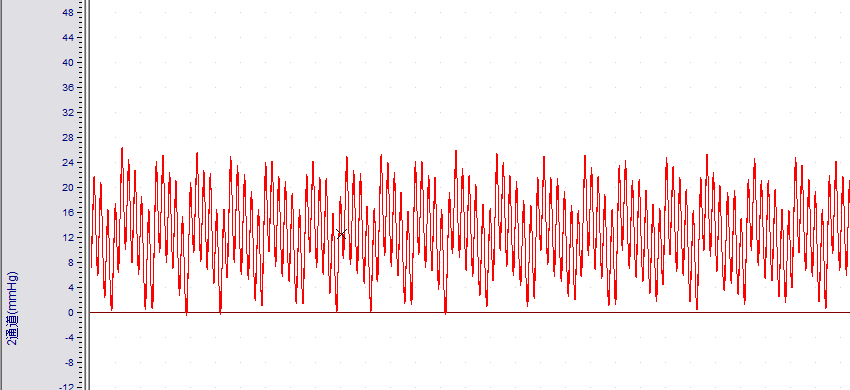

Supplement: S1 Raw data — (ZIP) [file pone.0235824.s002.zip › Raw data/Figure3 A1.png]

**Figure 5A**

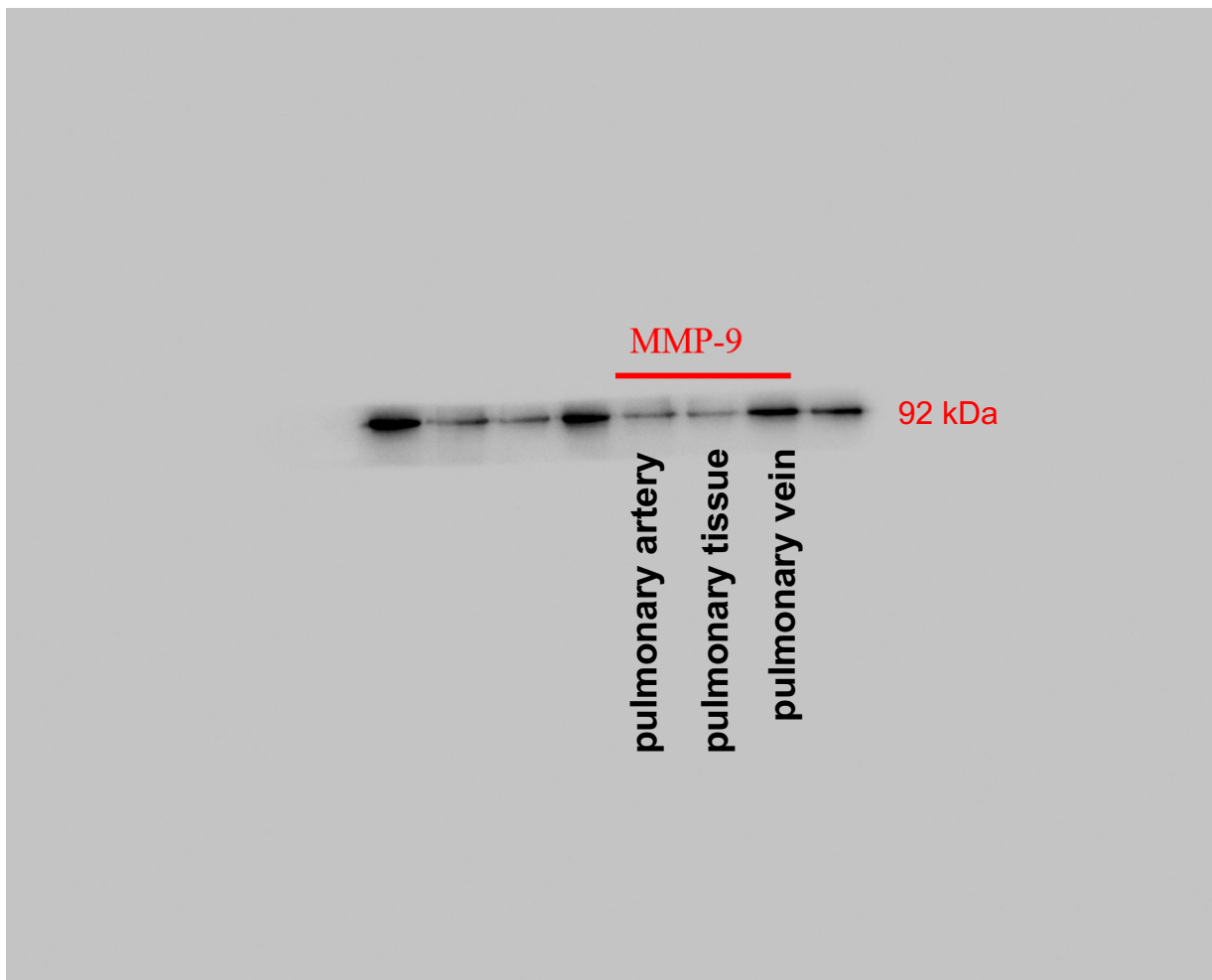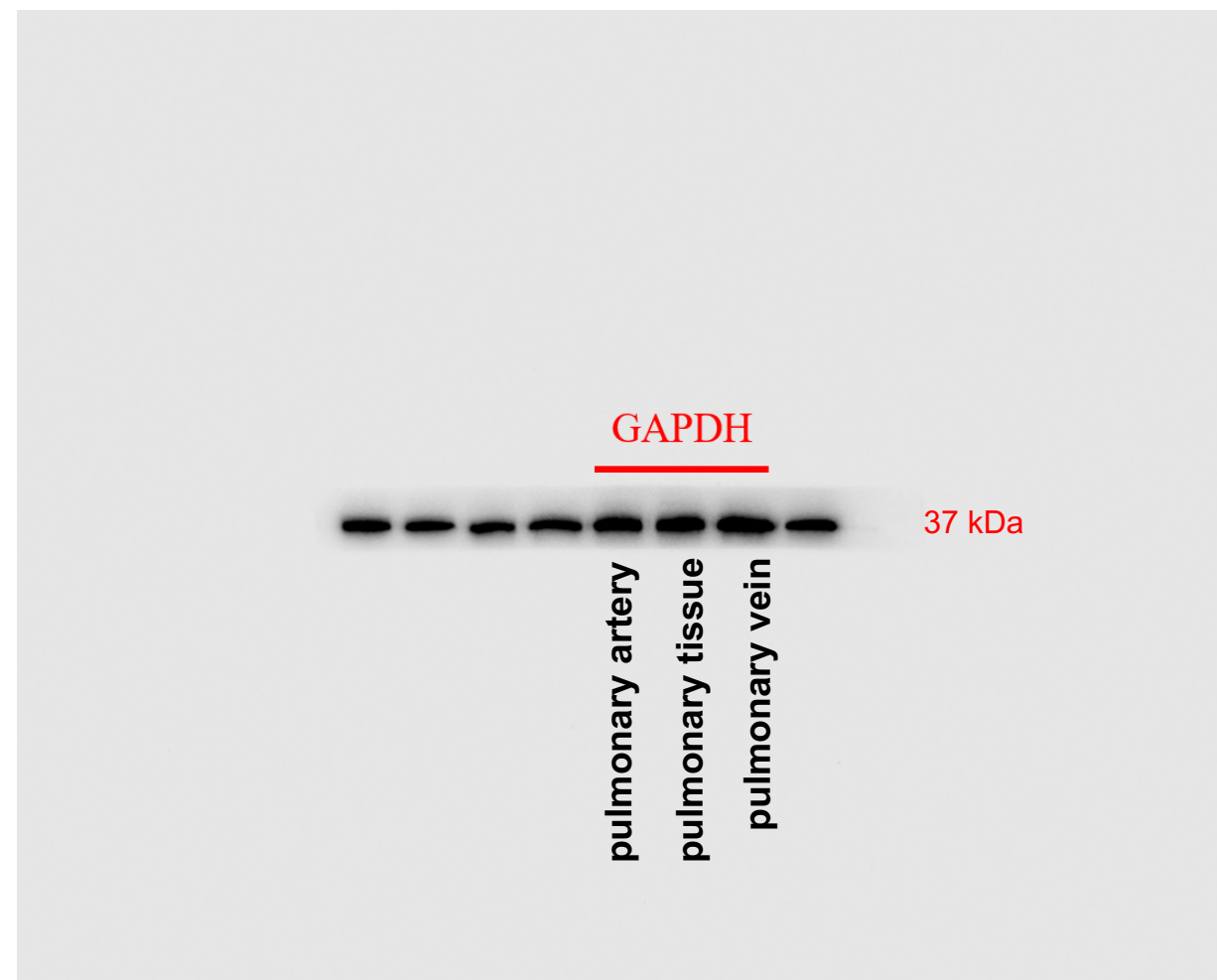

**Figure 5B**

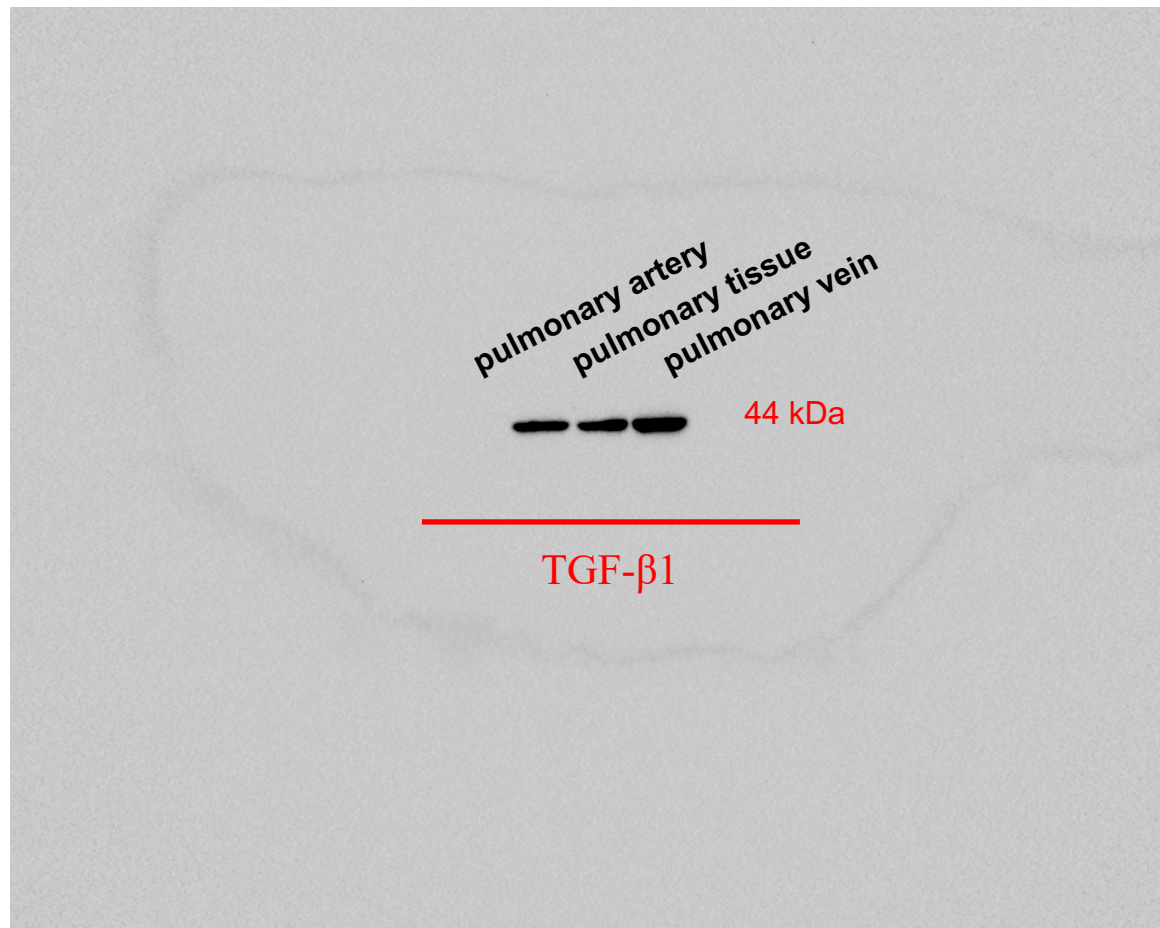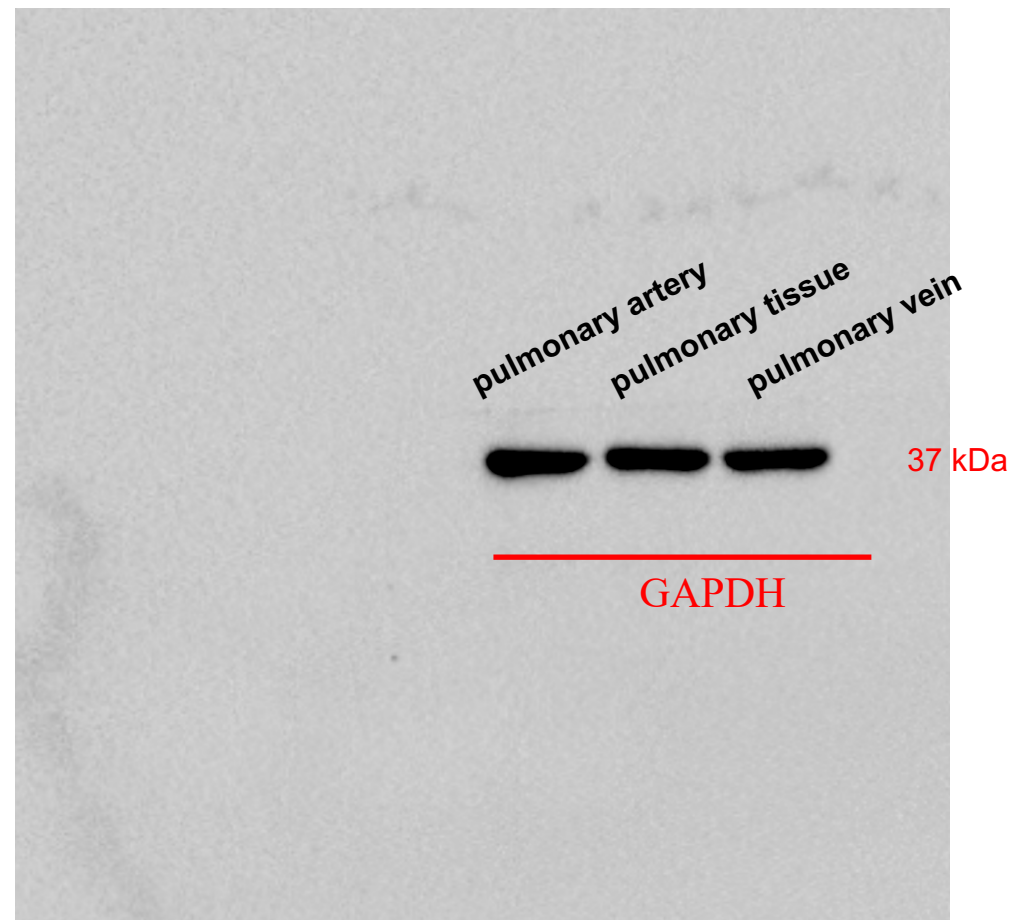

Figure 6C up

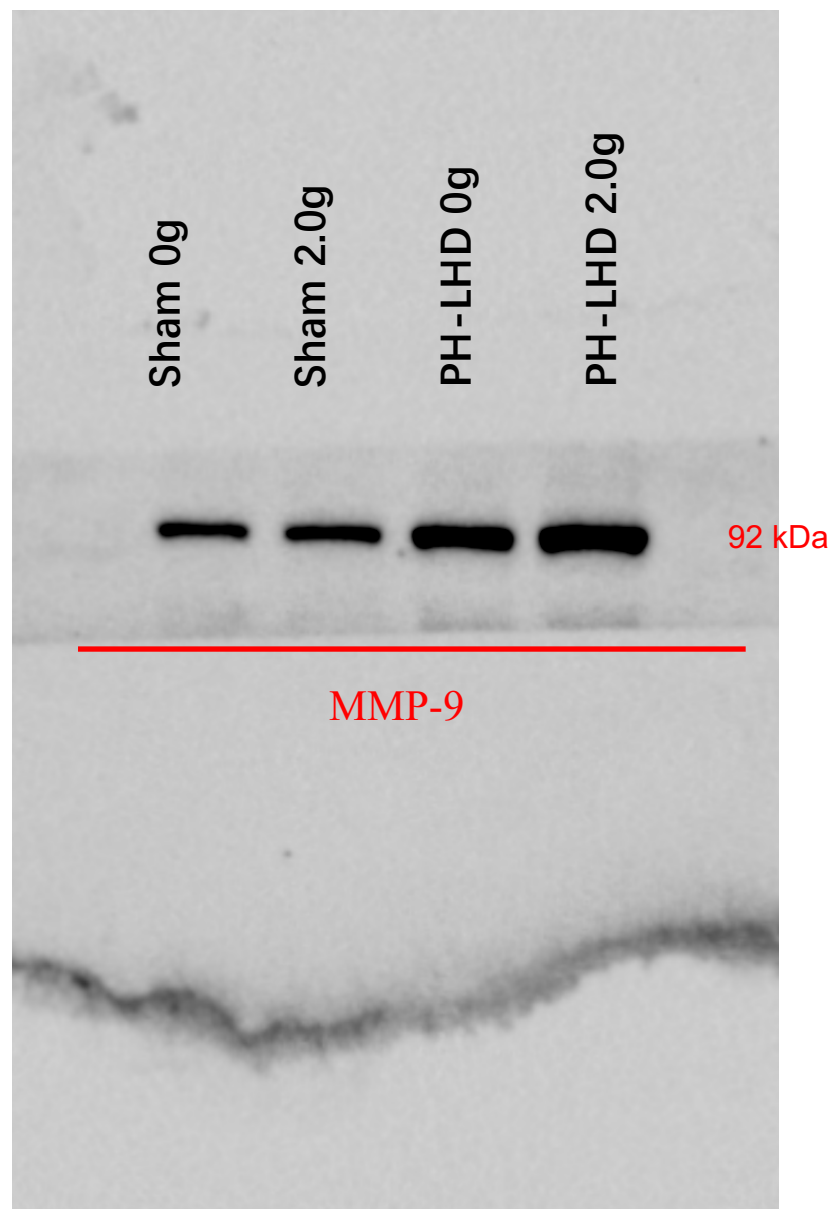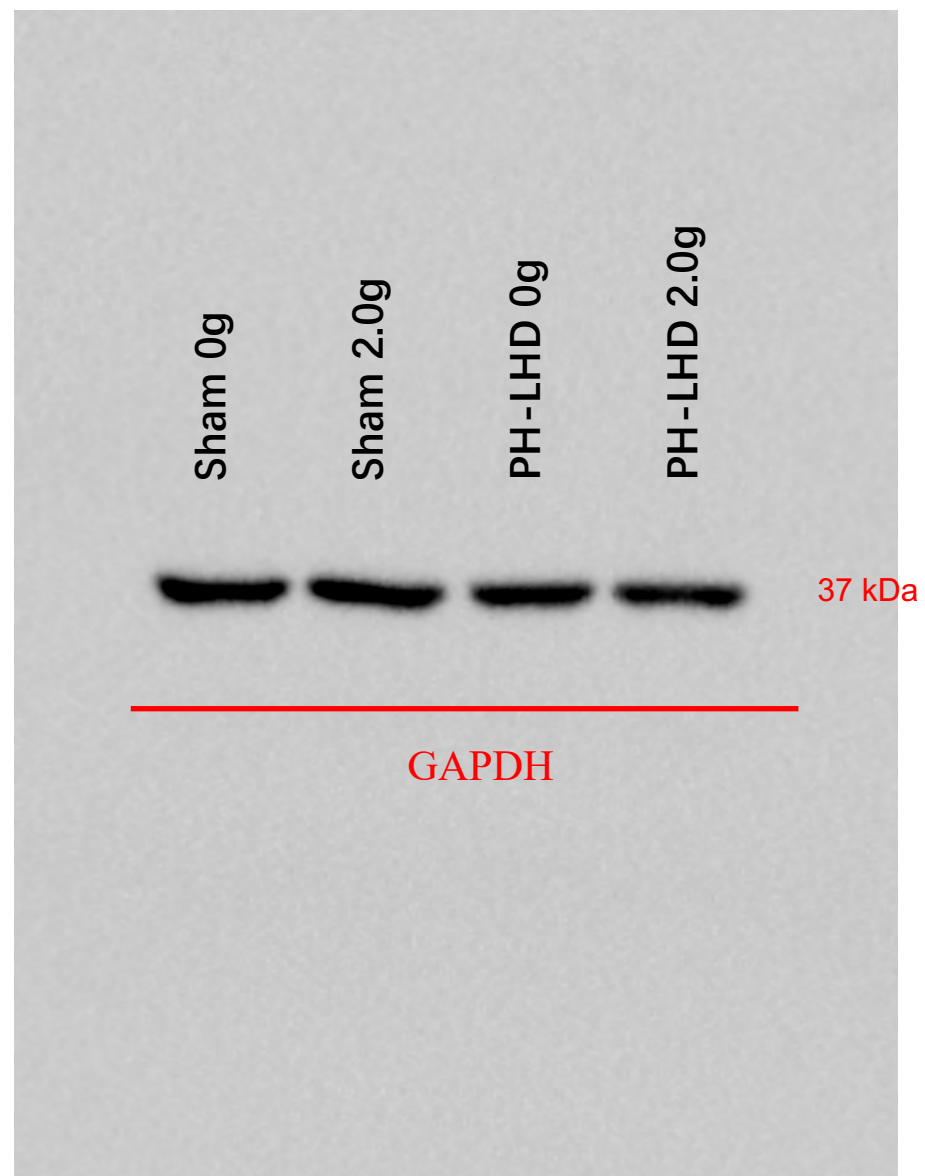

Figure 6C down

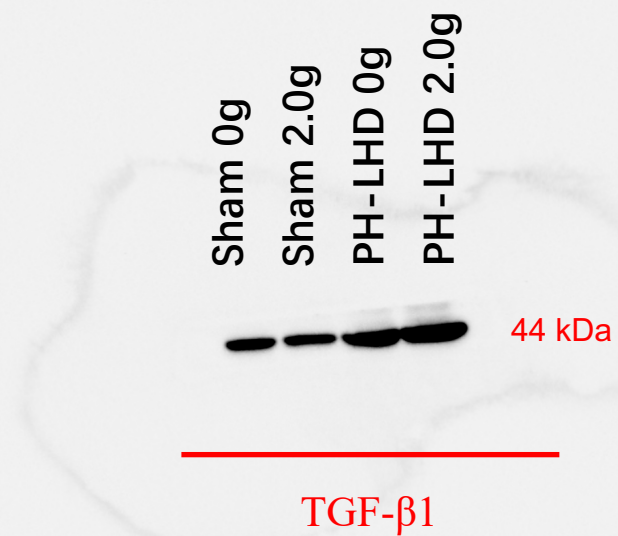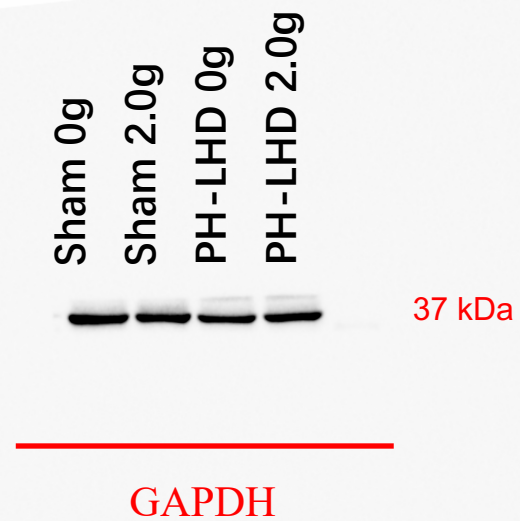

**Figure 7A**

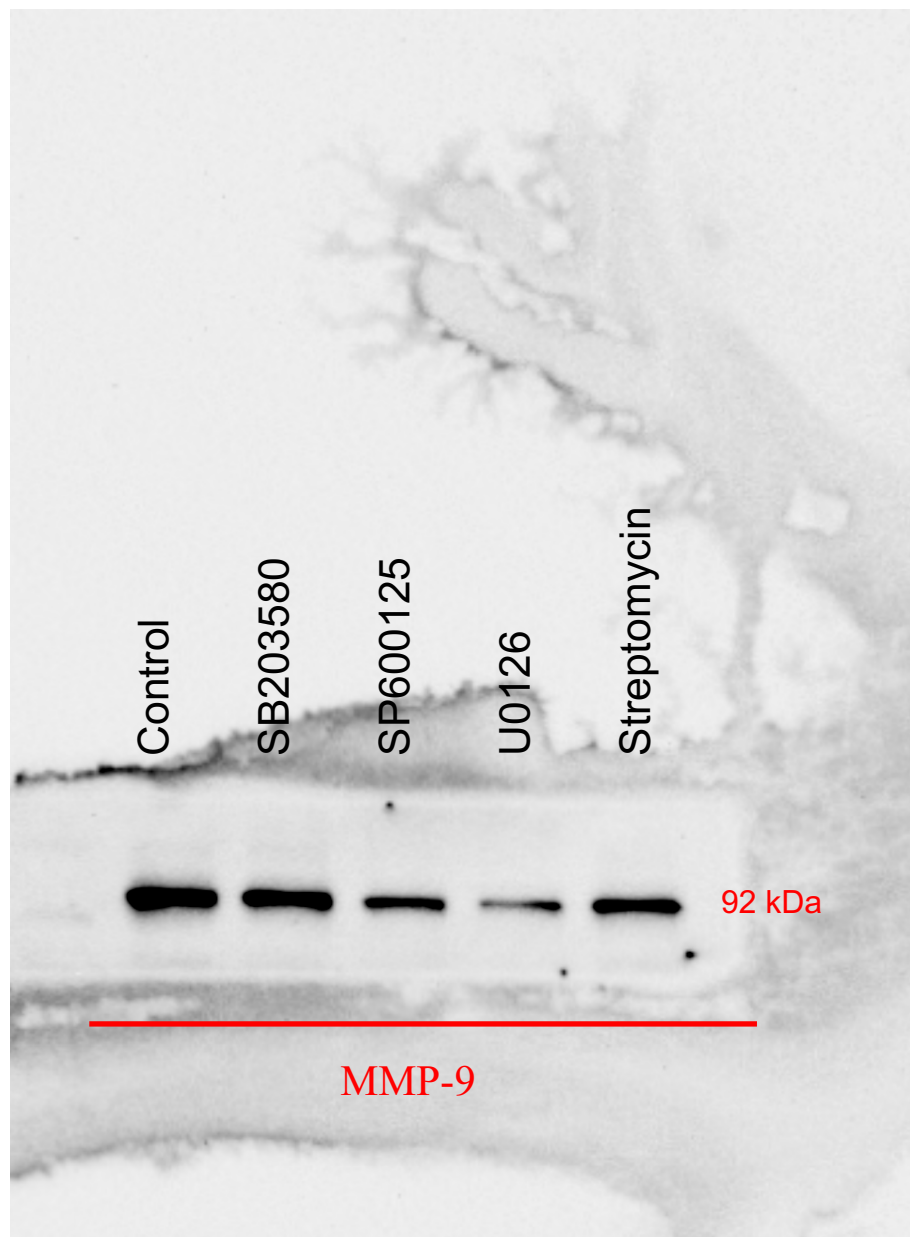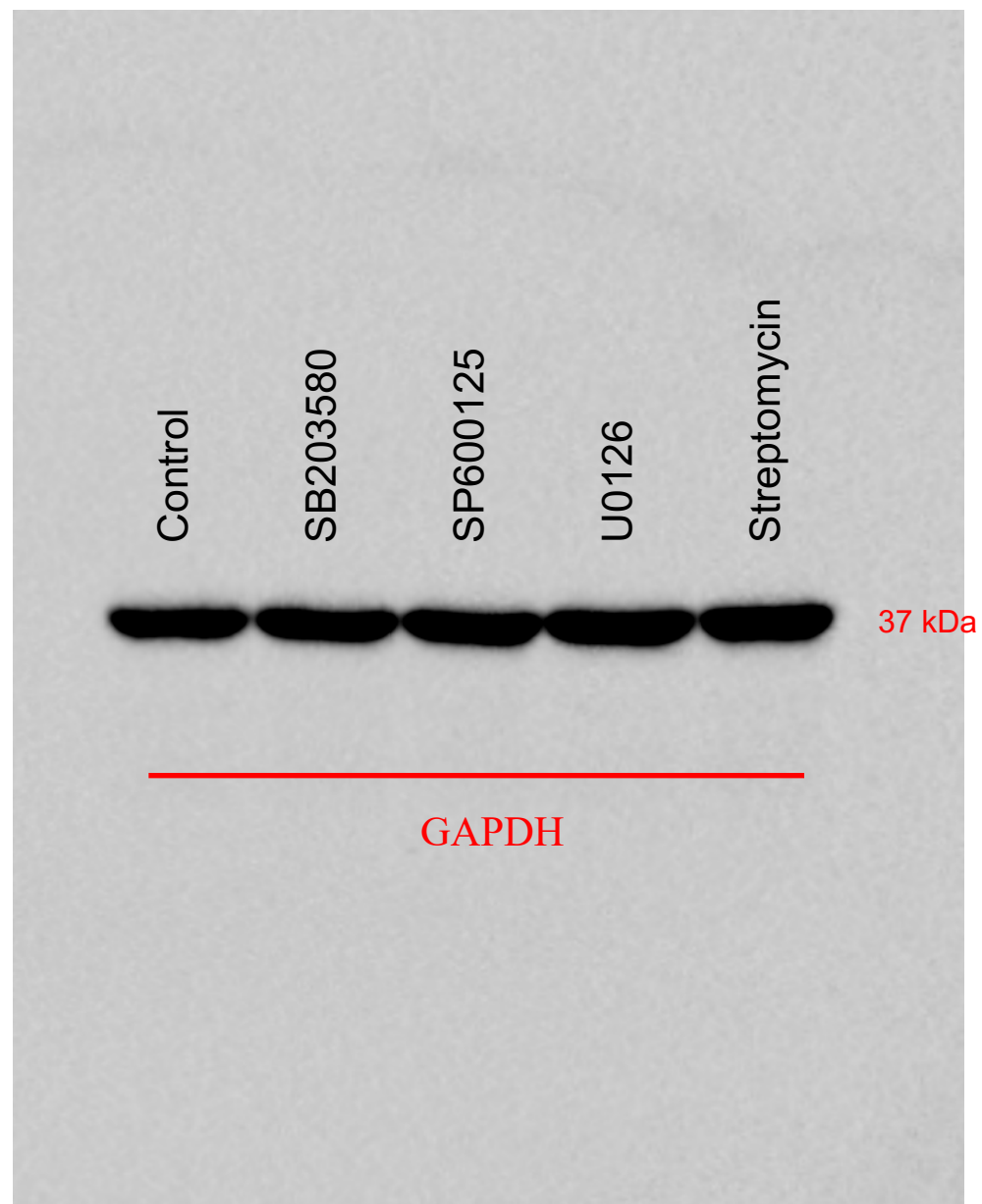

Figure 7B

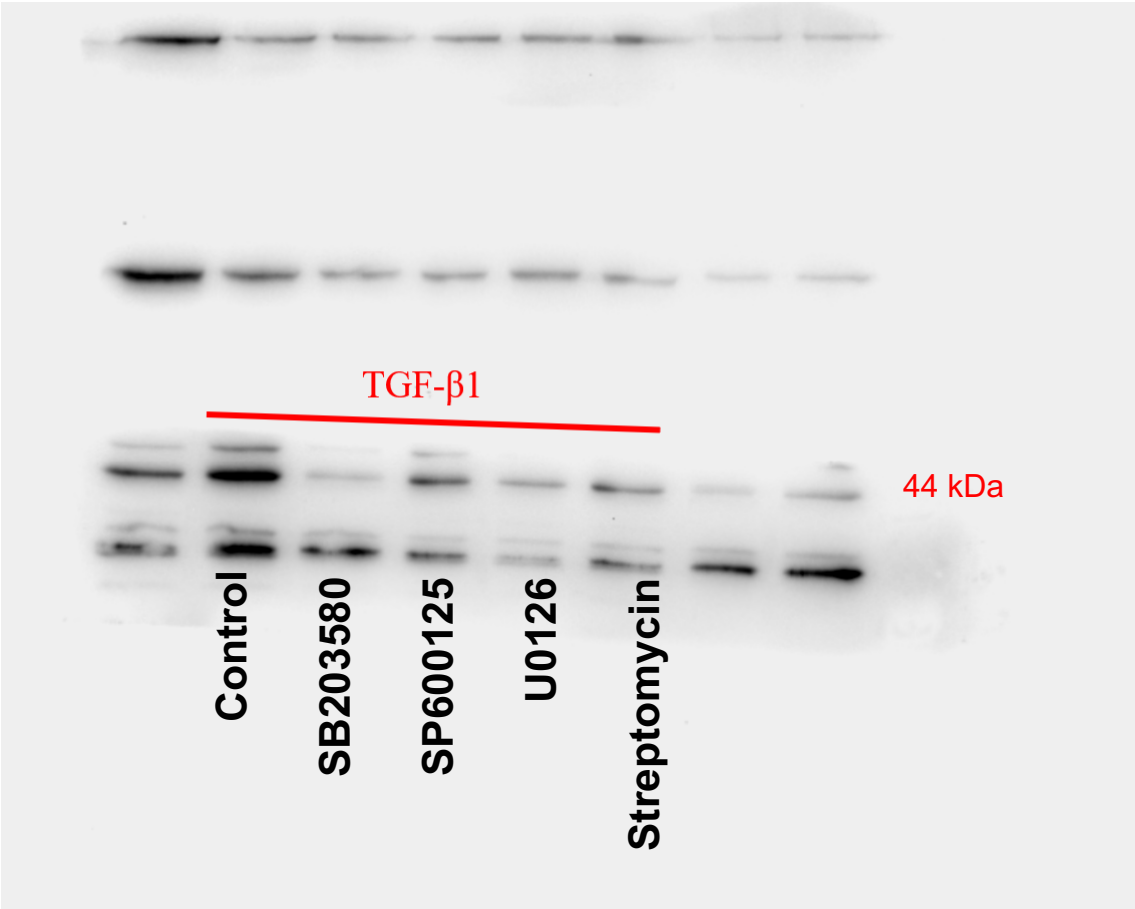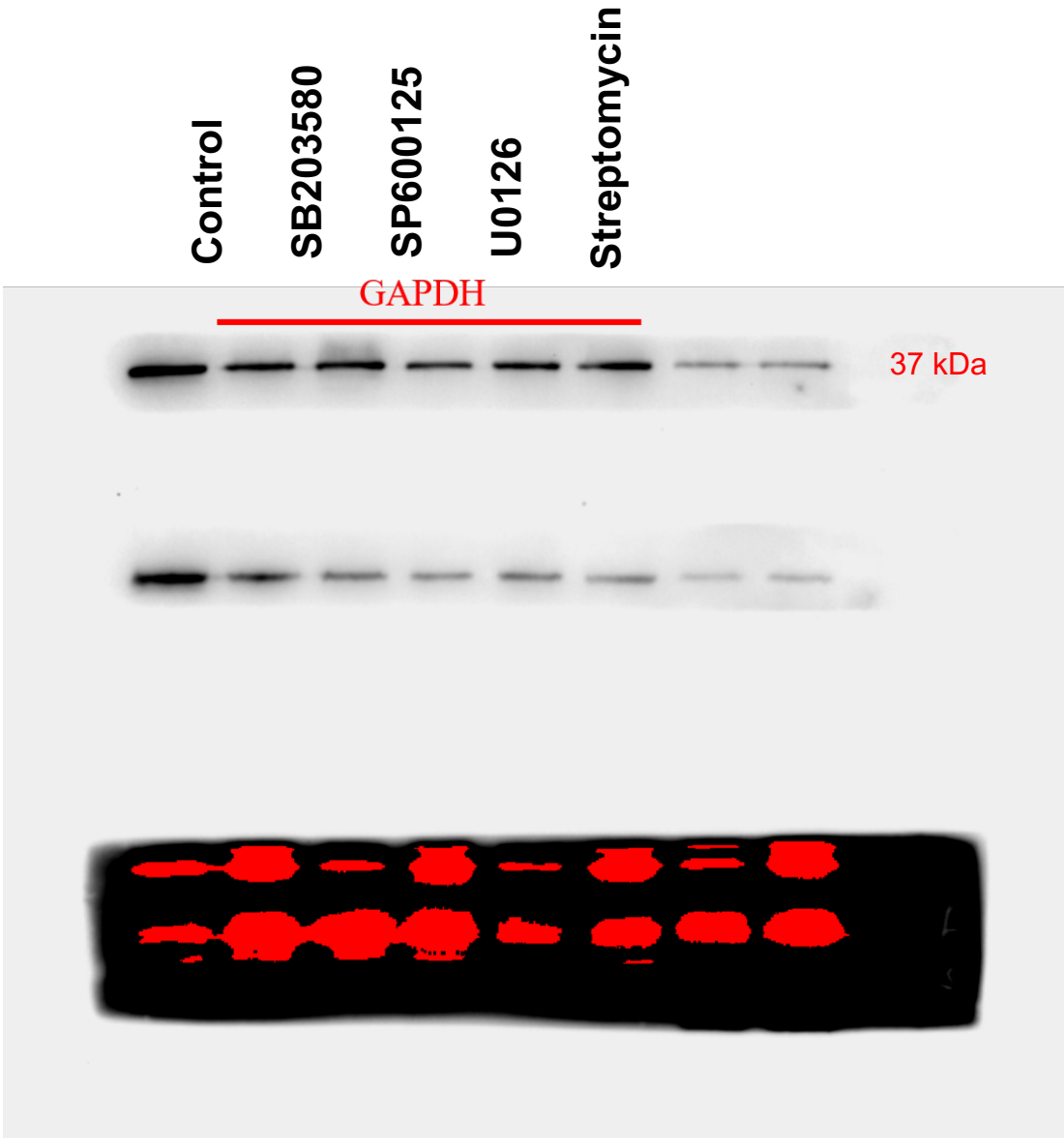

Supplement: S1 Raw images — (PDF) [file pone.0235824.s003.pdf]
