# Supplementary material for: Mechanical stretching of pulmonary vein stimulates matrix metalloproteinase-9 and transforming growth factor-β1 through stretch-activated channel/MAPK pathways in pulmonary hypertension due to left heart disease model rats
Source: PLoS One. 2020 Sep 3;15(9):e0235824. doi: 10.1371/journal.pone.0235824 (PMC7470280; doi:10.1371/journal.pone.0235824)
Supplement: Attachment — Submitted filename: Response to Reviewers-2.docx [file pone.0235824.s004.docx]

Dear reviewers:

We sincerely appreciate your time and critical comments on our manuscript entitled "Mechanical stretching of pulmonary vein stimulates matrix metalloproteinase-9 and transforming growth factor-β1 through stretch-activated channel/MAPK pathways in pulmonary hypertension due to left heart disease model rats" (PONE-D-19-35944). We have revised our manuscript as suggested and answered comments point by point.

--------------------------------------------------------------------

1) PLOS requires an ORCID iD for the corresponding author in Editorial Manager on papers submitted after December 6th, 2016. Please ensure that you have an ORCID iD and that it is validated in Editorial Manager. To do this, go to ‘Update my Information’ (in the upper left-hand corner of the main menu), and click on the Fetch/Validate link next to the ORCID field. This will take you to the ORCID site and allow you to create a new iD or authenticate a pre-existing iD in Editorial Manager. Please see the following video for instructions on linking an ORCID iD to your Editorial Manager account: https://www.youtube.com/watch?v=_xcclfuvtxQ
**Answer:** Thanks very much for your comments. We have created the ORCID iD.

2) PLOS ONE now requires that authors provide the original uncropped and unadjusted images underlying all blot or gel results reported in a submission’s figures or Supporting Information files. This policy and the journal’s other requirements for blot/gel reporting and figure preparation are described in detail at https://journals.plos.org/plosone/s/figures#loc-blot-and-gel-reporting-requirements and https://journals.plos.org/plosone/s/figures#loc-preparing-figures-from-image-files. When you submit your revised manuscript, please ensure that your figures adhere fully to these guidelines and provide the original underlying images for all blot or gel data reported in your submission. See the following link for instructions on providing the original image data: https://journals.plos.org/plosone/s/figures#loc-original-images-for-blots-and-gels.
**Answer:** Thanks very much for your comments. We have added the original uncropped and unadjusted images of all blots in our study.

3) Thank you for including your ethics statement:

All rats in this study have been approved by the Fujian Medical University. All animal experiments were in compliance with the National Institutes of Health Guide for Care and Use of Laboratory Animals.

Please amend your current ethics statement to confirm that your named ethics committee specifically approved this Study.

For additional information about PLOS ONE submissions requirements for ethics oversight of animal work, please refer to http://journals.plos.org/plosone/s/submission-guidelines#loc-animal-research  

Once you have amended this/these statement(s) in the Methods section of the manuscript, please add the same text to the “Ethics Statement” field of the submission form (via “Edit Submission”).

Authors should always state the organism(s) studied in the Abstract. Where the study may be confused as pertaining to clinical research, authors should also state the animal model in the title.

To maximize reproducibility and potential for re-use of data, we encourage authors to follow the [Animal Research: Reporting of In Vivo Experiments (ARRIVE) guidelines](http://www.nc3rs.org.uk/page.asp?id=1357) for all submissions describing laboratory-based animal research and to upload a completed [ARRIVE Guidelines Checklist](http://www.nc3rs.org.uk/page.asp?id=1357) to be published as supporting information.

**Answer:** Thanks very much for your comments. We have described the “Ethics Statement” in detail in the revised manuscript.

4) In your data availability statement, you have indicated that your data are available on request. Please note, PLOS ONE only allows data to be available upon request if there are specific ethical or legal restrictions on sharing the data publicly.  PLOS policy requires that all data required to replicate all study findings reported in the article be made publicly available, either as supporting information or in a public repository.

If there are no restrictions, please upload the minimal (anonymized) data set necessary to replicate your study findings to a stable, public repository and provide us with the relevant URLs, DOIs, or accession numbers. For a list of recommended repositories, please see our website (https://journals.plos.org/plosone/s/recommended-repositories).

If you are not able to upload the data to a repository, please upload the minimal (anonymized) data set as supporting information. For more information on different ways to make data available, please check our website here: https://journals.plos.org/plosone/s/data-availability#loc-acceptable-data-sharing-methods
**Answer:** Thanks very much for your comments. We have added the data as supporting information.

We appreciate very much for your time in reviewing our manuscript. And the suggestions and comments really helped us a lot. If there is any information I can provide, please don’t hesitate to contact us.

Thank you again for your time and patience. We are looking forward to hearing from you soon.

With kindest regards to you.

Yours sincerely
